# Modeling the Influence of Vaccine Administration on COVID-19 Testing Strategies

**DOI:** 10.3390/v13122546

**Published:** 2021-12-19

**Authors:** Jonathan E. Forde, Stanca M. Ciupe

**Affiliations:** 1 Department of Mathematics and Computer Sciences, Hobart and William Smith Colleges, Geneva, NY 14456, USA; 2 Department of Mathematics, Virginia Tech, Blacksburg, VA 24060, USA; stanca@vt.edu

**Keywords:** COVID-19 vaccination, Delta variant, mathematical modeling, testing, multi-scale models

## Abstract

Vaccination is considered the best strategy for limiting and eliminating the COVID-19 pandemic. The success of this strategy relies on the rate of vaccine deployment and acceptance across the globe. As these efforts are being conducted, the severe acute respiratory syndrome coronavirus 2 (SARS-CoV-2) is continuously mutating, which leads to the emergence of variants with increased transmissibility, virulence, and resistance to vaccines. One important question is whether surveillance testing is still needed in order to limit SARS-CoV-2 transmission in a vaccinated population. In this study, we developed a multi-scale mathematical model of SARS-CoV-2 transmission in a vaccinated population and used it to predict the role of testing in an outbreak with variants of increased transmissibility. We found that, for low transmissibility variants, testing was most effective when vaccination levels were low to moderate and its impact was diminished when vaccination levels were high. For high transmissibility variants, widespread vaccination was necessary in order for testing to have a significant impact on preventing outbreaks, with the impact of testing having maximum effects when focused on the non-vaccinated population.

## 1. Introduction

The emergence in 2019 of the novel acute respiratory syndrome coronavirus 2 (SARS-CoV-2) in Wuhan, China [1] has had devastating global consequences including loss of lives [2], strained healthcare systems [3,4,5], and economic recessions. Protective measures, such as masking, physical distancing, testing, contact tracing, quarantine, and isolation, while effective when applied rigorously, have proved insufficient in limiting the spread of SARS-CoV-2 [6,7,8]. Development of COVID-19 vaccines has been the main focus of public health organizations and pharmaceutical companies across the world [9,10,11].

The U.S. Food and Drug Administration (FDA) has approved two mRNA (Pfizer-BioNTech and Moderna) and one viral vector-based (Janssen) COVID-19 vaccines [12], which have consistently demonstrated effectiveness against disease and increased protection against infection [13,14,15,16]. Despite early control in highly vaccinated communities, vaccine shortage in low-access countries and vaccine hesitancy in high-access countries or regions, has led to selection of new variants [17], which might overcome vaccine-induced immunity [18,19].

Delta variant (B.1.617.2) was identified in the United States in March 2021 and accounted for 98.4% of new infections by 23 September 2021 [17]. It is believed to be more contagious than the ancestral SARS-CoV-2. One study quantified the mean basic reproductive number for the Delta variant to be $R_{0}=5.08$, compared to $R_{0}=2.79$ for the ancestral strain [20]. Another estimated that the Delta variant was 58–120% more transmissible than Alpha variant (B.1.1.7) across New England [21]. Recent studies measuring the effectiveness of vaccines against the transmission of the Delta variant found a reduction in the Pfizer-BioNTech effectiveness to 33.5% and 88% after one and two doses, compared to 49% and 94% effectiveness against the transmission of the Alpha variant [19]. CDC defines vaccine breakthrough infection as the detection of SARS-CoV-2 RNA or antigen in a respiratory specimen collected from a person ≥14 days after receipt of all recommended doses of an FDA-authorized COVID-19 vaccine [22]. The increased transmissibility of the Delta variant and the observed waning in vaccine effectiveness against infection highlight the critical importance of vaccinating an entire community and of offering boosters to individuals vaccinated at least six months prior [23]. With vaccines only recently approved for children aged 5 to 11, it is also important to adhere to rigorous COVID-19 prevention strategies such as masking and testing. One question of importance to the public health authorities, and the goal of this study, is to determine the best strategy for using testing in an increasingly vaccinated population in order to limit transmission.

SARS-CoV-2 diagnostic and surveillance testing is an important intervention tool for controlling transmission. While still widely used in monitoring the non-vaccinated population, little is known about the potential benefit of testing vaccinated population. Given limited resources, it is important to identify who to test in order to most effectively control transmission at minimal cost.

In this study, we developed a multi-scale mathematical model to examine the effect of testing on blocking SARS-CoV-2 infections in populations vaccinated with the Pfizer-BioNTech vaccine [24,25,26]. We expanded our previous work, which connected a within-host model for the time of SARS-CoV-2 infectiousness onset based on an individual virus dynamics and a between-host model for the transmission at the population level [24]. In particular, we incorporated a variable for the population vaccinated with the Pfizer-BioNTech vaccine and two populations of vaccinated individuals that become infected and have asymptomatic or symptomatic disease. We connected the vaccine effectiveness in preventing infection with the age of vaccination and the effect of the vaccine on reported individual virus profile when a breakthrough infection occurs [27]. We were interested in determining how vaccination level combined with surveillance testing can help reduce an infectious event with variants of various infectiousness level.

## 2. Materials and Methods

We model the interaction between a susceptible class S(t), vaccinated class v(t,α), infected class of asymptomatic individuals $i_{a}\left(\tau ,t\right)$, infected class of symptomatic individuals $i_{s}\left(\tau ,t\right)$, infected class of vaccinated asymptomatic individuals $i_{va}\left(\tau ,t\right)$, infected class of vaccinated symptomatic individuals $i_{vs}\left(\tau ,t\right)$, individuals recovered from natural infection R(t), individuals vaccinated after natural infection $R_{v1}\left(t\right)$ and individuals recovered after being vaccinated and then infected $R_{v2}\left(t\right)$. The independent variables are the age of infection in an individual τ, the age of vaccination in an individual α, and the time-since-outbreak in the population *t*. The parameters are the transmission rate β, the infection weighting functions $\lambda_{j}$, the birth rate *b*, the death rate μ, the disease-induced mortality rate *m*, the vaccination rate ν, and the degree of protection after vaccine administration η. Moreover, we consider testing in both vaccinated and non-vaccinated populations, with a testing capacity of *C* tests per day, leading to case detection rates $r_{j}$, with j∈{a,s,va,vs}.

As in our earlier study [24], we assume that an individual’s infection status is given by its virus profile at age of infection τ. In particular, given virus profiles for infected individuals, we link test positivity to the ages of infection during which virus load is above the sensitivity threshold. Similarly, we determine the ages of infection during which the virus load is high enough to allow transmission. We define

$$\begin{array}{cc} \tau_{1}^{j} & =\mathrm{age}\;\mathrm{for}\;\mathrm{onset}\;\mathrm{of}\;\mathrm{virus}\;\mathrm{detectability}\;\mathrm{by}\;\mathrm{PCR}\;\mathrm{test},\\ \tau_{2}^{j} & =\mathrm{age}\;\mathrm{for}\;\mathrm{onset}\;\mathrm{of}\;\mathrm{infectiousness},\\ \tau_{3}^{j} & =\mathrm{age}\;\mathrm{for}\;\mathrm{end}\;\mathrm{of}\;\mathrm{infectiousness},\\ \tau_{4}^{j} & =\mathrm{age}\;\mathrm{for}\;\mathrm{end}\;\mathrm{of}\;\mathrm{virus}\;\mathrm{detectability}\;\mathrm{by}\;\mathrm{PCR}\;\mathrm{test}, \end{array}$$

where j∈{a,s,va,vs}. The infectivity weighting functions $\lambda_{j}$ are

$$\begin{array}{cc} \lambda_{a}\left(\tau \right) & =\left\{\begin{array}{cc} \gamma , & \mathrm{for}\tau \in [\tau_{2}^{a},\tau_{3}^{a}]\\ 0, & \mathrm{otherwise} \end{array}\right.,\\ \lambda_{s}\left(\tau \right) & =\left\{\begin{array}{cc} 1, & \mathrm{for}\tau \in [\tau_{2}^{s},\tau_{3}^{s}]\\ 0, & \mathrm{otherwise} \end{array}\right.,\\ \lambda_{va}\left(\tau \right) & =\left\{\begin{array}{cc} \gamma , & \mathrm{for}\tau \in [\tau_{2}^{va},\tau_{3}^{va}]\\ 0, & \mathrm{otherwise} \end{array}\right.,\\ \lambda_{vs}\left(\tau \right) & =\left\{\begin{array}{cc} 1, & \mathrm{for}\tau \in [\tau_{2}^{vs},\tau_{3}^{vs}]\\ 0, & \mathrm{otherwise} \end{array}\right., \end{array}$$

where parameter 0<γ<1 represents the relative infectiousness of asymptomatic carriers, in comparison with symptomatic carriers [22]. For all infected classes, $0\leq \tau \leq \tau_{q}$. For $\tau >\tau_{q}$ infections are considered resolved, and recovered individual are not susceptible to reinfection.

**Daily testing rate.** As before [24], we define a daily per capita testing rate, ρ, corresponding to an overall testing capacity of *C* tests per day at time *t* in a population of size N(t) to be given by

$$\rho =-\ln\left(1-\frac{C}{N(t)}\right),$$

with C<N(t). When testing is administered to only non-vaccinated individuals,

$$N\left(t\right)=S\left(t\right)+R\left(t\right)+\int_{0}^{\tau_{q}}\left[i_{a}\left(\tau ,t\right)+i_{s}\left(\tau ,t\right)\right]d\tau ,$$

and the case detection rate functions $r_{j}\left(\tau ,t\right)$ become

$$r_{j}\left(\tau ,t\right)=\left\{\begin{array}{cc} \rho (t), & \mathrm{for}t\geq 0\mathrm{and}\tau_{1}^{j}\leq \tau \leq \tau_{4}^{j}\\ 0, & \mathrm{otherwise} \end{array}\right.,$$

for j∈{a,s}, and $r_{va}\left(\tau ,t\right)=r_{vs}\left(\tau ,t\right)=0$ for all τ.

When testing is administered to both vaccinated and non-vaccinated individuals,

$$N\left(t\right)=S\left(t\right)+R\left(t\right)+R_{v1}\left(t\right)+R_{v2}\left(t\right)+\int_{0}^{\tau_{q}}\left[i_{a}\left(\tau ,t\right)+i_{s}\left(\tau ,t\right)+i_{va}\left(\tau ,t\right)+i_{vs}\left(\tau ,t\right)\right]d\tau ,$$

and the case detection rate functions $r_{j}\left(\tau ,t\right)$ become

$$r_{j}\left(\tau ,t\right)=\left\{\begin{array}{cc} \rho (t), & \mathrm{for}t\geq 0\mathrm{and}\tau_{1}^{j}\leq \tau \leq \tau_{4}^{j}\\ 0, & \mathrm{otherwise} \end{array}\right.,$$

where j∈{a,s,va,vs}. We assume a test return delay of *ℓ* days and that individuals who receive a positive test result are isolated, and can no longer transmit the virus. Lastly, we ignore the possibility of reinfection.

**Model equations.** On the domain t≥0, $0\leq \tau \leq \tau_{q}$, and α≥0, the between-host model equations under combined vaccination and testing are

(1) $$\begin{array}{cc} \frac{dS}{dt} & =b-\mu S-\nu S-\beta S(t)\Lambda (t),\\ \frac{\partial v}{\partial \alpha }+\frac{\partial v}{\partial t} & =-\mu v(\alpha ,t)-\beta (1-\eta (\alpha ))v(\alpha ,t)\Lambda (t),\\ \frac{\partial i_{a}}{\partial \tau }+\frac{\partial i_{a}}{\partial t} & =-\left(\mu +m\right)i_{a}\left(\tau ,t\right)-r_{a}\left(\tau_{\ell },t_{\ell }\right)i_{a}\left(\tau_{\ell },t_{\ell }\right)e^{-(\mu +m)\ell },\\ \frac{\partial i_{s}}{\partial \tau }+\frac{\partial i_{s}}{\partial t} & =-\left(\mu +m\right)i_{s}\left(\tau ,t\right)-r_{s}\left(\tau_{\ell },t_{\ell }\right)i_{s}\left(\tau_{\ell },t_{\ell }\right)e^{-(\mu +m)\ell },\\ \frac{\partial i_{va}}{\partial \tau }+\frac{\partial i_{va}}{\partial t} & =-\left(\mu +m\right)i_{va}\left(\tau ,t\right)-r_{va}\left(\tau_{\ell },t_{\ell }\right)i_{va}\left(\tau_{\ell },t_{\ell }\right)e^{-(\mu +m)\ell },\\ \frac{\partial i_{vs}}{\partial \tau }+\frac{\partial i_{vs}}{\partial t} & =-\left(\mu +m\right)i_{vs}\left(\tau ,t\right)-r_{vs}\left(\tau_{\ell },t_{\ell }\right)i_{vs}\left(\tau_{\ell },t_{\ell }\right)e^{-(\mu +m)\ell },\\ \frac{dR}{dt} & =-\mu R-\nu R+i_{a}\left(\tau_{q},t\right)+i_{s}\left(\tau_{q},t\right)\\ & \;+\int_{0}^{\tau_{q}}\left[r_{a}\left(\tau_{\ell },t_{\ell }\right)i_{a}\left(\tau_{\ell },t_{\ell }\right)e^{-(\mu +m)\ell }+r_{s}\left(\tau_{\ell },t_{\ell }\right)i_{s}\left(\tau_{\ell },t_{\ell }\right)e^{-(\mu +m)\ell }\right]d\tau ,\\ \frac{dR_{v1}}{dt} & =-\mu R_{v1}+\nu R,\\ \frac{dR_{v2}}{dt} & =-\mu R_{v2}+i_{va}\left(\tau_{q},t\right)+i_{vs}\left(\tau_{q},t\right)\\ & \;+\int_{0}^{\tau_{q}}\left[r_{va}\left(\tau_{\ell },t_{\ell }\right)i_{va}\left(\tau_{\ell },t_{\ell }\right)e^{-(\mu +m)\ell }+r_{vs}\left(\tau_{\ell },t_{\ell }\right)i_{vs}\left(\tau_{\ell },t_{\ell }\right)e^{-(\mu +m)\ell }\right]d\tau . \end{array}$$

where

(2) $$\Lambda \left(t\right)=\int_{0}^{\tau_{q}}\left[\lambda_{a}\left(\tau \right)i_{a}\left(\tau ,t\right)+\lambda_{s}\left(\tau \right)i_{s}\left(\tau ,t\right)+\lambda_{va}\left(\tau \right)i_{va}\left(\tau ,t\right)+\lambda_{vs}\left(\tau \right)i_{vs}\left(\tau ,t\right)\right]d\tau$$

is the weighted infectious population. The subscript *ℓ* represents a delay of *ℓ* days, so $\tau_{\ell }=\tau -\ell$, $t_{\ell }=t-\ell$.

The initial conditions are

(3) $$\begin{array}{cc} S(0) & =S_{0}=\frac{b}{\mu }-V_{0}-I_{0},\\ v(\alpha ,0) & =V_{0}\delta \left(\alpha -\alpha_{f}\right),\\ i_{a}\left(\tau ,0\right) & =\left(1-f\right)I_{0}\delta \left(\tau \right),\\ i_{s}\left(\tau ,0\right) & =fI_{0}\delta \left(\tau \right),\\ i_{va}\left(\tau ,0\right) & =0,\\ i_{vs}\left(\tau ,0\right) & =0,\\ R(0) & =0,\\ R_{v1}\left(0\right) & =0,\\ R_{v2}\left(0\right) & =0. \end{array}$$

The boundary conditions are

(4) $$\begin{array}{cc} v(0,t) & =\nu S(t),\\ i_{a}\left(0,t\right) & =\left(1-f\right)\beta \Lambda \left(t\right)\left[S\left(t\right)+\int_{0}^{\alpha_{v}}\left[1-\eta (\alpha )\right]v\left(\alpha ,t\right)d\alpha \right],\\ i_{s}\left(0,t\right) & =f\beta \Lambda \left(t\right)\left[S\left(t\right)+\int_{0}^{\alpha_{v}}\left[1-\eta (\alpha )\right]v\left(\alpha ,t\right)d\alpha \right],\\ i_{va}\left(0,t\right) & =\left(1-f\right)\beta \Lambda \left(t\right)\int_{\alpha_{v}}^{\infty }\left[1-\eta (\alpha )\right]v\left(\alpha ,t\right)d\alpha ,\\ i_{vs}\left(0,t\right) & =f\beta \Lambda \left(t\right)\int_{\alpha_{v}}^{\infty }\left[1-\eta (\alpha )\right]v\left(\alpha ,t\right)d\alpha , \end{array}$$

where *f* is the fraction of infections that are symptomatic, $\alpha_{v}$ is the age of vaccination after which an individual’s virus load is reduced if infected, and $\alpha_{f}$ is the age of vaccination after which vaccines provide full protection. For $\alpha <\alpha_{v}$, vaccinated individuals are equivalent to susceptible individuals from the point of view of their virus profile, but they are less likely to get infected. Parameters $\left\{\beta ,\mu ,m,f,\alpha_{v}\right\}$ are taken from literature, and δ is the Dirac delta function.

### 2.1. Cumulative Statistics

In order to compare model results with commonly tabulated public health data, we define several cumulative population statistics derived from the model state variables.

The cumulative number of cases up to time *t*, Σ(t), is given by the equation

(5) $$\Sigma \left(t\right)=I_{0}+\int_{0}^{t}\left[i_{a}\left(0,s\right)+i_{s}\left(0,s\right)+i_{va}\left(0,s\right)+i_{vs}\left(0,s\right)\right]ds.$$

The cumulative number of positive case detections at time *t*, P(t), is given by the equation

(6) $$\begin{array}{cc} \frac{dP}{dt} & =\int_{0}^{\infty }\left[r_{a}\left(\tau_{\ell },t_{\ell }\right)i_{a}\left(\tau_{\ell },t_{\ell }\right)e^{-(\mu +m)\ell }+r_{s}\left(\tau_{\ell },t_{\ell }\right)i_{s}\left(\tau_{\ell },t_{\ell }\right)e^{-(\mu +m)\ell }\right.\\ & \;\left.+r_{va}\left(\tau_{\ell },t_{\ell }\right)i_{va}\left(\tau_{\ell },t_{\ell }\right)e^{-(\mu +m)\ell }+r_{vs}\left(\tau_{\ell },t_{\ell }\right)i_{vs}\left(\tau_{\ell },t_{\ell }\right)e^{-(\mu +m)\ell }\right]d\tau , \end{array}$$

with initial condition P(0)=0.

The cumulative number of SARS-CoV-2 naive individuals who have reached full vaccination status by time *t* is given by

(7) $$F\left(t\right)=V_{0}+\int_{0}^{t}v\left(\alpha_{f},s\right)ds,$$

and the cumulative number of previously infected individuals reaching full vaccination status by time *t* is given by

(8) $$CVR\left(t\right)=\int_{0}^{t}\nu R\left(s-\alpha_{f}\right)e^{-\mu \alpha_{f}}ds.$$

Thus, the cumulative number of fully vaccinated individuals at time *t* is

(9) T(t)=F(t)+CVR(t).

Breakthrough cases are infections of fully vaccinated individuals, so the cumulative number of breakthrough cases up to time *t*, B(t), is the number of new infections occurring in individuals with $\alpha \geq \alpha_{f}$. B(t) is given by the equation

(10) $$\frac{dB}{dt}=\beta \Lambda \left(t\right)\int_{\alpha_{f}}^{\infty }\left[1-\eta (\alpha )\right]v\left(\alpha ,t\right)d\alpha ,$$

with initial condition B(0)=0.

### 2.2. Parameter Choices

We parametrize our model based on efficacy data from Pfizer-BioNTech vaccine against the Alpha and Delta variants [19,28]. Therefore, we set the rate 0≤η(α)≤1 describing the degree of protection against the Alpha variant α days after vaccine administration to

(11) $$\eta \left(\alpha \right)=\left\{\begin{array}{cc} 0, & \mathrm{for}\;\alpha <7,\\ 0.49, & \mathrm{for}\;7\leq \alpha <\alpha_{f},\\ 0.94, & \mathrm{for}\;\alpha \geq \alpha_{f}, \end{array}\right.$$

and against Delta variant, α days after vaccine administration, to

(12) $$\eta \left(\alpha \right)=\left\{\begin{array}{cc} 0, & \mathrm{for}\;\alpha <7,\\ 0.33, & \mathrm{for}\;7\leq \alpha <\alpha_{f},\\ 0.88, & \mathrm{for}\;\alpha \geq \alpha_{f}. \end{array}\right.$$

**Figure 1:**
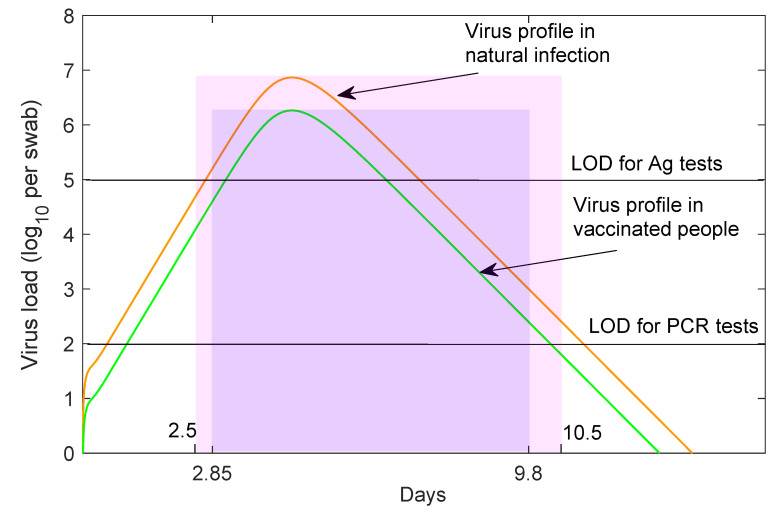
Virus profiles in non-vaccinated and vaccinated individuals. $\log_{10}$ virus load per swab over time during Alpha variant natural infection (red line) and vaccination (green line) as given by the within-host model in [29]. Non-vaccinated patients are assumed to be infectious from t=2.5 days till t=10.5 days (shaded pink region). Vaccinated patients are assumed to be infectious from t=2.85 days till t=9.8 days (shaded purple region). Black horizontal lines correspond to RT-PCR test detection threshold (LOD) of $\log_{10}\left(V\right)=2$ per swab and antigen test detection threshold (LOD) of $\log_{10}\left(V\right)=5$ per swab.

The CDC defines a fully vaccinated individual as one who is ≥14 days past receipt of all recommended doses of an FDA-authorized vaccine [22]. In our model, this corresponds to $\alpha \geq \alpha_{f}=35$ days. Levine et al. have shown that, for the first 11 days following the Pfizer-BioNTech vaccination, the cycle threshold (CT) values in infected vaccinated individuals do not change compared to those of infected yet non-vaccinated individuals [27]. We therefore set $\alpha_{v}=11$ days. Afterwards, virus levels decrease by four-fold in vaccinated individuals [27]. The reduction in virus load leads to both shorter infectivity period, ranging between $\tau_{2}^{j}=2.8$ and $\tau_{3}^{j}=9.8$ days post infection, and shorter time for detection by tests, ranging between $\tau_{1}^{j}=0.97$ and $\tau_{4}^{j}=10.22$ days post infection (j={va,vs}) for RT-PCR (see Figure 1). We assume a PCR test return delay of ℓ=1 days.

We assume that the Alpha variant transmissibility rate is β=0.25, as in our previous study [24], and the Delta variant transmissibility rate is 1.6-times higher, β=0.4, in agreement with recent studies [20,21]. The other parameters $\left\{\mu ,m,f,\tau_{q}\right\}$ are as in our previous study [24]. A summary of parameters is given in Table 1.

## 3. Results

### 3.1. Alpha Variant Dynamics in the Absence of Testing

**Figure 2:**
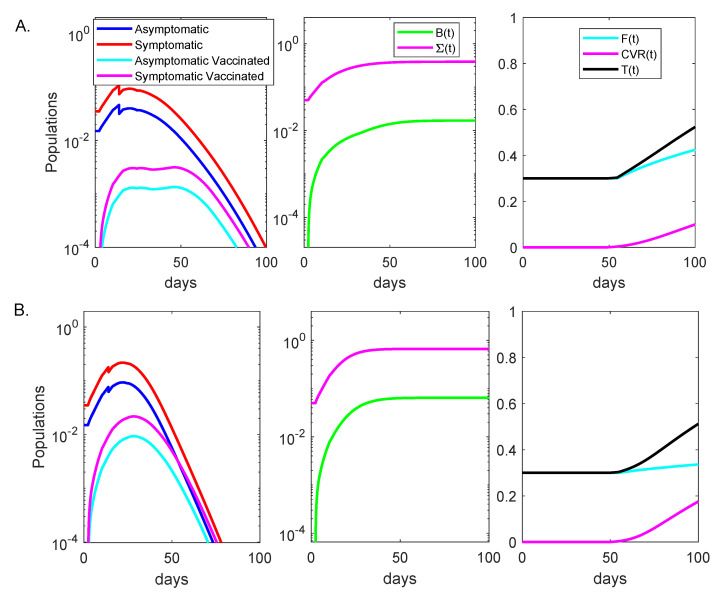
Dynamics of Alpha and Delta variants infection over time. Left panels: daily asymptomatic (blue), symptomatic (red), breakthrough asymptomatic (cyan), and breakthrough asymptomatic (magenta) infections over time; Middle panels: cumulative cases Σ(t) (magenta), cumulative breakthrough cases B(t) (green) over time; Right panels: cumulative fully vaccinated F(t) (cyan), cumulative vaccinated after infection CVR(t) (magenta) and cumulative total vaccinated T(t) (black) over time in the absence of testing. Panel (**A**): Alpha variant; Panel (**B**): Delta variant. The background vaccination is 30% and the other parameters and initial conditions are given in Table 1.

To simulate an initially undetected outbreak in a partially vaccinated population, we assumed that at time $t_{0}=0$, when a total of $V\left(0\right)=V_{0}=30\%$ of individuals have been fully vaccinated, $I_{0}=5\%$ of the population was infected with the Alpha variant, f=70% with symptomatic and 1−f=30% with asymptomatic disease. The vaccine efficacy was given by (11), ν=1% additional vaccines were administered daily starting with day $t_{1}=20$, and no testing was considered. Daily symptomatic, asymptomatic, breakthrough asymptomatic and breakthrough symptomatic cases peaked at 10.6%, 4.6%, 0.31% and 0.13% of the entire population at days 14, 14, 47 and 47, respectively (see Figure 2A, left panel, red, blue, magenta and cyan lines). At day 100, a cumulative $\Sigma_{noTests}\left(100\right)=38.4\%$ of the population had been infected when not fully vaccinated (see Figure 2A, middle panel, magenta line) and a cumulative B(100)=1.69% of the population had been infected while fully vaccinated (see Figure 2A, middle panel, green line). Lastly, at day 100 a cumulative F(100)=42% of the naive population has been fully vaccinated (see Figure 2A, right panel, cyan line), a cumulative CVR(100)=9.8% of the population had been fully vaccinated after recovering from natural infection (see Figure 2A, right panel, magenta line), and a cumulative T(100)=52% of the population had been vaccinated (see Figure 2, right panel, black line). A quantity of interest is the percent breakthrough number, B(100)/F(100), defined as the percent of cumulative naive fully vaccinated individuals who get infected divided by the cumulative fully vaccinated population. In this case, we obtained a percent breakthrough case rate B(100)/F(100)=4% at day 100.

**Figure 3:**
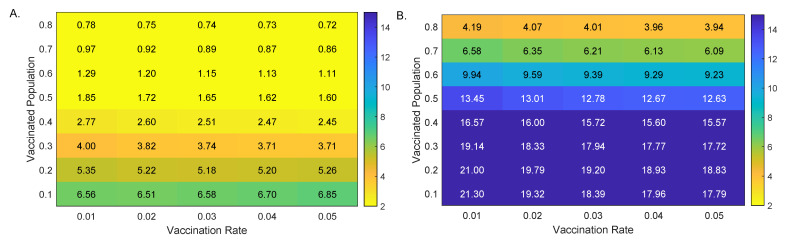
Percent breakthrough cases at day 100. Heatmaps for the percent breakthrough cases in the vaccinated population at day 100 versus additional daily vaccines, ν, and background vaccination levels, $V_{0}$. Panel (**A**): Alpha variant; Panel (**B**): Delta variant. Parameters and initial conditions are given in Table 1.

To more closely determine the relationship between the percent breakthrough cases at day 100, B(100)/F(100), the vaccination level at the start of the outbreak, $V_{0}$, and daily vaccination level starting at day 20, ν, we derived a heat map for the percent breakthrough cases for smaller $V_{0}$ and ν increments (see Figure 3A). We observed that the percent breakthrough cases range between 0.72% for $V_{0}=80\%$ and ν=5% and 6.56% for $V_{0}=10\%$ and ν=1% (see Figure 3A). Having a large percent of the population vaccinated at the time of the outbreak resulted in a decrease in the proportion of fully vaccinated individuals experiencing breakthrough cases.

### 3.2. Alpha Variant Model Outcomes in the Presence of Testing

We investigated the effect of fixed daily testing with capacity *C*, administered beginning at day $t_{1}=20$ in two cases. Case 1: only the non-vaccinated group was tested; Case 2: both the vaccinated and non-vaccinated groups were tested. In particular, we quantified the effects of RT-PCR tests with return delay of one day in reducing total infections 100 days after the outbreak, $\Sigma_{noTest}\left(100\right)-\Sigma_{Test}\left(100\right)$.

**Figure 4:**
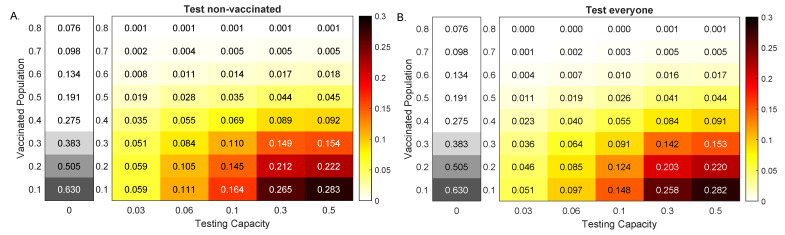
Reduction in Alpha variant cases at 100 days. Heatmaps for the reduction in cumulative cases at 100 days after an outbreak with an Alpha variant, $\Sigma_{noTests}\left(100\right)-\Sigma_{Tests}\left(100\right)$, as given by model Equation (1) versus RT-PCR testing capacity with a return delay of 1 day, *C*, and background vaccination levels, $V_{0}$. Panel (**A**): Test non-vaccinated only; Panel (**B**): Test everybody. The gray heatmaps represent the cumulative cases at day 100 in the absence of testing, $\Sigma_{noTest}\left(100\right)$. Parameters and initial conditions are given in Table 1.

For low vaccinated population, increased testing resulted in increased reduction in cumulative cases, regardless of the testing strategy. In particular, for $V_{0}=10\%$ vaccinated population and no testing, the outbreak resulted in $\Sigma_{noTests}\left(100\right)=63\%$ infections by day 100 (see Figure 4, grey heatmaps). When testing only non-vaccinated individuals, this number was reduced by 5.9, 16.4 and 28.3 percentage points for C=0.03, C=0.1 and C=0.5, respectively (see Figure 4A, colored heatmaps). When testing everyone, this number was reduced by 5.1, 14.8 and 28.2 percentage points for C=0.03, C=0.1 and C=0.5, respectively (see Figure 4B, colored heatmaps).

For high vaccinated population, increased testing had no effect on cumulative cases, regardless of the testing strategy. In particular, for $V_{0}=80\%$ vaccinated population and no testing, the outbreak resulted in $\Sigma_{noTests}\left(100\right)=7.6\%$ infections by day 100 (see Figure 4, grey heatmaps). Testing affected this number by 0.1 percentage points for C≥0.03 when testing non-vaccinated individuals and for C≥0.3 when testing everyone, respectively (see Figure 4, colored heatmaps).

Note that, for both testing scenarios, the amount of reduction decreased with the increase of $V_{0}$ (for a fixed ν) and increased with the increase of testing capacity *C* (for a fixed $V_{0}$). Moreover, the highest decrease happened for the highest testing in the least vaccinated population. Lastly, in all cases, the model predicted that testing only non-vaccinated results in improved outcomes.

### 3.3. Delta Variant Dynamics in the Absence of Testing

We examined the effect of the Pfizer–BioNTech vaccine on blocking infections with the Delta variant. To account for the increased infectiousness of Delta, we increased the transmissibility rate to β=0.4, 1.6-times higher than in the case of the Alpha variant. Moreover, we modified the Pfizer–BioNTech effectiveness to (12).

As before, we simulated an initially undetected outbreak in a partially vaccinated population, with $V_{0}=30\%$ of the population fully vaccinated at the time of the outbreak, ν=1% additional vaccines were administered daily starting with day $t_{1}=20$, and no testing was considered. Daily symptomatic, asymptomatic, breakthrough symptomatic and breakthrough asymptomatic cases peaked at 21.7%, 9.2%, 2.2% and 0.9% of the entire population at days 22, 22, 29 and 29, respectively (see Figure 2B, left panel, red, blue, magenta and cyan lines). At day 100, a cumulative $\Sigma_{noTests}\left(100\right)=66.7\%$ of the population had been infected when not fully vaccinated (see Figure 2B, middle panel, magenta line) and a cumulative B(100)=6.44% of the population has been infected while fully vaccinated (see Figure 2B, middle panel, green line). Lastly, at day 100, the model predicted that a cumulative F(100)=33.5% of the naive population had been fully vaccinated (see Figure 2B, right panel, cyan line), a cumulative CVR(100)=17.2% of the population had been fully vaccinated after recovering from natural infection (see Figure 2B, right panel, magenta line), and a cumulative T(100)=51% of the population had been vaccinated (see Figure 2B, right panel, black line). The percent breakthrough case rate at day 100 became B(100)/F(100)=19.22%.

When we expanded our analysis to include smaller $V_{0}$ and ν increments, the model prediction for breakthrough cases at day 100 increased to B(100)/F(100)=21.3% (compared to 6.56% for the Alpha variant) for $V_{0}=10\%$ and ν=1% and to B(100)/F(100)=3.94% (compared to 0.72% for the Alpha variant) for $V_{0}=80\%$ and ν=5% (see Figure 3B). For this more contagious variant, having a large percent of the population vaccinated at the time of the outbreak significantly decreased the breakthrough cases.

### 3.4. Delta Variant Model Outcomes in the Presence of Testing

We next investigated the effect of fixed daily testing with capacity *C*, administered beginning at day $t_{1}=20$ in reducing total infections 100 days after the outbreak, $\Sigma_{noTest}\left(100\right)-\Sigma_{Test}\left(100\right)$, when only the non-vaccinated and when everyone was tested.

**Figure 5:**
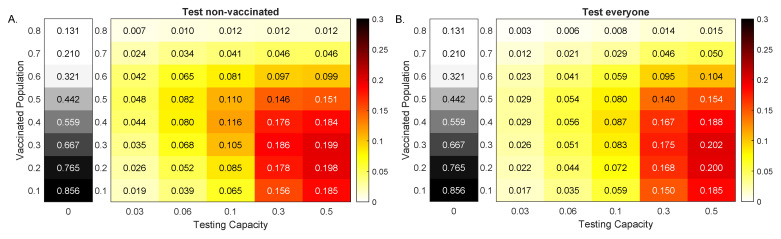
Reduction in Delta variant cases at 100 days. Heatmaps for the reduction in cumulative cases at 100 days after an outbreak with a Delta variant, $\Sigma_{noTests}\left(100\right)-\Sigma_{Tests}\left(100\right)$, as given by model Equation (1) versus RT-PCR testing capacity with a return delay of 1 day, *C*, and background vaccination levels, $V_{0}$. Panel (**A**): Test non-vaccinated only; Panel (**B**): Test everybody. The gray heatmaps represent the cumulative cases at day 100 in the absence of testing, $\Sigma_{noTest}\left(100\right)$. Parameters and initial conditions are given in Table 1.

When less than 60% of the population was vaccinated at the time of the outbreak, increased testing led to increased reduction in cumulative cases, regardless of the testing strategy. In particular, for $V_{0}=10\%$ vaccinated population and no testing, the outbreak resulted in $\Sigma_{noTest}\left(100\right)=85.6\%$ of the population being infected by day 100 (see Figure 5, grey heatmaps). When testing non-vaccinated individuals only, this number was reduced by 1.9, 6.5 and 18.5 percentage points for C=0.03, C=0.1 and C=0.5, respectively (see Figure 5A, colored heatmaps). When testing everyone, this number was reduced by 1.7, 5.9 and 18.5 percentage points for C=0.03, C=0.1 and C=0.5, respectively (see Figure 5B, colored heatmaps). For $V_{0}=30\%$ and no testing, the outbreak resulted in $\Sigma_{noTest}\left(100\right)=66.7\%$ of the population being infected by day 100 (see Figure 5, grey heatmaps). When testing non-vaccinated individuals only, this number was reduced by 3.5, 10.5 and 19.9 percentage points for C=0.03, C=0.1 and C=0.5, respectively (see Figure 5A, colored heatmaps). When testing everyone, this number was reduced by 2.6, 8.3 and 20.2 percentage points for C=0.03, C=0.1 and C=0.5, respectively (see Figure 5B, colored heatmaps). Finally, for $V_{0}=60\%$ vaccinated population and no testing, the outbreak resulted in $\Sigma_{noTest}\left(100\right)=32.1\%$ of the population being infected by day 100 (see Figure 5, grey heatmaps). When testing only non-vaccinated individuals, this number was reduced by 4.2, 8.1 and 9.9 percentage points for C=0.03, C=0.1 and C=0.5, respectively (see Figure 5A, colored heatmaps). When testing everyone, this number was reduced by 2.3, 5.9 and 10.4 percentage points for C=0.03, C=0.1 and C=0.5, respectively (see Figure 5B, colored heatmaps). As with the Alpha variant, the amount of reduction increased with the increase of *C* (for a fixed $V_{0}$), regardless of the testing scenarios. Unlike the Alpha variant case, however, the reduction did not decrease monotonically with the increase of $V_{0}$ (for fixed *C*). The maximum reduction occured at different $V_{0}$ values for different testing capacities *C*. In particular, for both testing strategies, the maximum reductions occured at $V_{0}=40\%$ for C=0.1 and $V_{0}=30\%$ for C=0.3.

When more than 60% of the population was vaccinated, increased testing resulted in increased reduction in cumulative cases when we tested everyone, but not when we only tested the non-vaccinated. That is why, for high vaccinated populations, testing everyone led to a slight increase in case reductions, compared with testing only the non-vaccinated.

### 3.5. The Role of Testing for Emerging Variant Dynamics

In an ongoing and evolving pandemic, the specific characteristics of the virus and of human behavior are unknown. Hence, the parameters upon which our model relies are uncertain. We therefore investigated the role of testing in reducing cases when we assumed outbreaks with variants of increased infectiousness. First, we considered populations of different vaccination levels, where vaccines maintained high efficacy rates (11), and second, we considered a 50% vaccinated population where variants induce decreased vaccines efficacy. In both cases we assumed a fixed testing capacity of C=0.1 with a PCR test with a return delay of one day.

**Figure 6:**
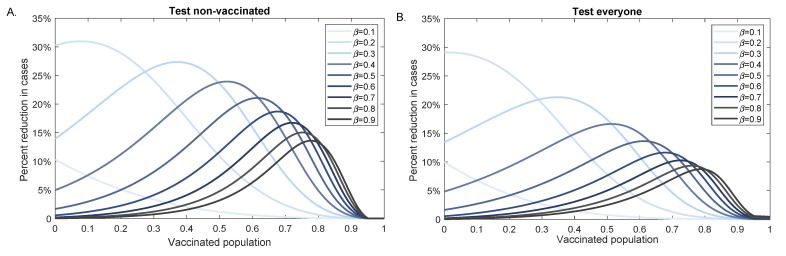
Percent reduction in variant cases at 100 days as vaccination increases. Percent reduction in cumulative cases at 100 days after an outbreak with a variants of different infectivity rate β, $\left(\Sigma_{noTests}\left(100\right)-\Sigma_{Tests}\left(100\right)\right)/\Sigma_{noTests}\left(100\right)$, as given by model Equation (1) versus background vaccination levels, $V_{0}$. Panel (**A**): Test non-vaccinated only; Panel (**B**): Test everybody. Parameters and initial conditions are given in Table 1 and we assume RT-PCR testing capacity C=0.1 with a return delay of 1 day.

When the vaccine efficacy is unchanged by increased variant infectiousness, testing led to higher case reduction for intermediate vaccination levels. The peak of the reduction decreased and shifted to the right as the infectiousness level increased (see Figure 6). For example, for high infectivity rate β=0.9 we obtained maximum testing effect for a population with 80% background vaccination level, while for a lower infectivity rate β=0.3 we obtained maximum testing effect for a population with 40% background vaccination level. The percent case reduction was higher when we tested only the non-vaccinated population but the peak reduction occured at the same background vaccination level, regardless of the testing strategy (see Figure 6A,B).

**Figure 7:**
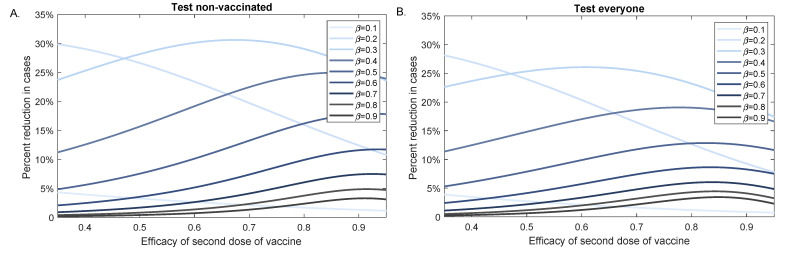
Percent reduction in variant cases at 100 days as vaccination efficacy decreases. Percent reduction in cumulative cases at 100 days after an outbreak with a variants of different infectivity rate β, $\left(\Sigma_{noTests}\left(100\right)-\Sigma_{Tests}\left(100\right)\right)/\Sigma_{noTests}\left(100\right)$, as given by model Equation (1) versus efficacy of the second dose of the vaccine, $\eta_{2}$. Panel (**A**): Test non-vaccinated only; Panel (**B**): Test everybody. Parameters and initial conditions are given in Table 1, we assume background vaccination $V_{0}=0.5$ and RT-PCR testing capacity C=0.1 with a return delay of 1 day.

We next investigated the effect of testing in reducing infectious cases when the vaccine efficacy decreased. We fixed the background vaccination level to $V_{0}=0.5$ and determined the percent case reduction for different β values when the efficacy of the second dose varied between $\eta_{2}=0.35$ and $\eta_{2}=0.95$ (see Figure 7). For β values for which $R_{0}<1$, the effect of testing decreased with increasing vaccine efficacy. For β values for which $R_{0}>1$, the effect of testing increased as vaccine efficacy increased. For $R_{0}>1$, testing had maximum effect for variants with $R_{0}$ close to 1 (β=0.2,0.3), regardless of testing strategy (see Figure 7A,B), and testing non-vaccinated individuals alone only marginally improved the outcomes.

## 4. Discussion

Reaching herd-immunity to SARS-CoV-2 through vaccination and natural infection is made harder by vaccine hesitancy, vaccine shortage around the world, emergence of new variants, and delayed vaccination approval for children aged 5 to 11 years. Therefore, additional public health interventions such as masks, social distancing, and surveillance testing are still needed. In previous work, we have used mathematical models to show that surveillance testing can be an effective public health intervention to reduce a SARS-CoV-2 outbreak in a non-vaccinated population [24]. The significance of surveillance testing needs to be re-evaluated in the context of vaccination, emergence of new variants, and waning immunity. CDC recommends that vaccinated people are tested only when experiencing COVID-19 symptoms or after coming into close contact with someone with COVID-19 [33]. To better determine the role of surveillance tests in a rapidly changing environment, we developed a multi-scale mathematical model of COVID-19 transmission in a mixed vaccinated and non-vaccinated population. Our model investigated how vaccination levels impact the effectiveness of testing. We compared two testing strategies: one in which tests were administered to both vaccinated and non-vaccinated individuals and one in which tests were administered to non-vaccinated individuals alone. Additionally, we considered variants of increased infectiousness.

In the case of variants with low transmission rates, such as the Alpha variant, where vaccines are highly effective in blocking transmission, we found that testing remained an effective intervention when the overall vaccination is low to moderate. For higher vaccination levels, the impact of testing was diminished, even relative to the smaller outbreaks that occured in those scenarios. For any fixed testing capacity, the number of cases prevented by testing decreased with increasing vaccination level.

For variants with higher transmission rates, such as the Delta variant, where vaccine efficacy in blocking transmission was reduced, a more complex pattern of testing effectiveness was apparent. For low vaccination, the impact of testing was low, as testing was not sufficient to overcome the force of infection created by the Delta variant. This difference to the case of the Alpha variant was a result of the magnitude of the outbreak, which was driven by increased transmissibility and the reduced effectiveness of the vaccine against the Delta variant. Interestingly, as the vaccination level increased, the number of cases prevented by testing increased as well, even though the number of cases that would occur in the absence of testing declined. As vaccination level increased further, the effectiveness of a fixed testing capacity declined again, due to the significant reduction in the number of cases expected in the absence of testing, as in the case of the Alpha variant. Thus, when considering the Delta variant, the impact of a fixed testing capacity was highest for moderate vaccination level and lower for low and high vaccination level.

We investigated differences in testing strategies. In the United States, it is common for surveillance testing to focus on the non-vaccinated population [33]. Here we compared the differences in outcomes for testing strategies that included only the non-vaccinated and those that included both vaccinated and non-vaccinated populations. We found that testing strategies that focused on the non-vaccinated population were generally more effective than broad testing strategies. For the Alpha variant, this was true for all cases considered. For the Delta variant, a broad testing strategy was preferable when the testing capacity exceeded the non-vaccinated population, a result that has been reported in other modeling studies [34]. This indicated that the most effective strategy should focus first on testing the non-vaccinated population, then using excess capacity in the non-vaccinated population.

As expected, for fixed vaccination level, increasing testing capacity increased the number of cases prevented. However, for high vaccination level, the impact of additional testing eventually saturated, and further testing had little or no effect.

We have also studied the prevalence of infection within the fully vaccinated population, so-called “breakthrough” cases. For the Alpha variant, the prevalence of breakthrough cases was uniformly low (ranging between 0.7% and 6.45%), and decreased as the population vaccination level increased. These results are similar to those from clinical studies which reported an Alpha variant incidence of breakthrough infections of 0.5% in health care workers in US who received either the Pfizer–BioNTech or the Moderna vaccine [35], of 2.6% in health care workers in Israel who received both doses of Pfizer–BioNTech vaccine [36], and of 0.08% in New York metropolitan area vaccinated with either Pfizer–BioNTech, Moderna, or Janssen vaccines [37]. For the Delta variant, our model predicted that breakthrough cases were much more prevalent in the vaccinated population and ranged between 3.94% and 21.3%. These numbers are comparable with the 8.4% breakthrough cases reported in Houston hospitals [38] and lower than the 28% reported in the Washington, DC area [39]. While the increased transmissibility of the Delta variant and the decreased effectiveness of vaccine were necessary conditions for this increase in breakthrough cases, the primary driving factor was the extent of the outbreak in the non-vaccinated population. As the vaccinated population became larger, both the total number of breakthrough cases and their prevalence decreased.

The results for the Alpha and Delta variants presented in this study were based on uncertain parameters. To better describe the dependence of our model predictions on evolving viral parameters, we quantified percent case reductions due to testing for variants of increased infectiousness and for variants that make vaccines less effective in preventing infection. We found that increased variant transmission rates (which may be due to the evolution of inherent viral characteristics influencing infectiousness or leading to higher viral load, or due to changing compliance with public health interventions) resulted in decreased testing efficacy. Interestingly, as variant transmission rates increased, testing was most useful when applied to populations with high vaccination background. The decrease in testing effectiveness can be compensated by increasing the testing frequency and decreasing the delay in results return (not shown). Applying these additional measures is especially important when variants of higher infectiousness become dominant, as they lead to larger and faster spreading outbreaks.

Similarly, we found that increased variant transmission rates resulted in decreased testing efficacy, regardless of the efficacy of the second dose of vaccine. The impact of second dose vaccine efficacy was less pronounced than the impact of the background vaccination rate. This indicates that even if vaccine efficacy wanes over time in individuals, maintaining a high vaccination rate in the population is an important factor in ensuring the effectiveness of testing strategies for outbreak reduction. As with the Alpha and Delta scenarios, focusing testing on non-vaccinated individuals resulted in improved outcomes, regardless of transmission rate or vaccine efficacy.

Our modeling approach includes several simplifying assumptions, some of which can be relaxed to generalize our results in a variety of ways. First, it is important to note that, in this study, the measure for the effectiveness of testing is the prevention of transmission, not the prevention of disease, hospitalization, or death. The true extent of transmission is generally not known in an ongoing outbreak, even when testing is widespread. Public health outcomes and vaccine effectiveness are generally expressed in terms of preventing disease, with all three vaccines approved for use in the United States being highly effective in preventing hospitalization and deaths regardless of the variant [40]. While preventing disease is the immediate goal, we argue that preventing transmission even when a large fraction of the population has been vaccinated (through surveillance testing) is a worthy long-term goal that may allow emergent variant strains resistant to vaccination to go extinct before becoming the next dominant strain [41]. Secondly, we assumed that a positive test return results in the complete isolation of the infected individual, who can no longer transmit the virus. This is a best case scenario, and further work is needed to incorporate other metrics, such as compliance rate.

We have started the outbreak with a large number of infectious individuals. When we decreased that initial burden from 5% to 1%, the epidemic curve flattened and the time to the peak lengthened. As a result, the effect of testing in lower vaccinated populations increased significantly (not shown). As before, the effect of testing in the highly vaccinated population saturated. This indicates that early detection of nascent outbreaks is essential for the effectiveness of testing as a public health intervention.

Our study is limited to PCR testing with a return delay of one day. In the past, we have investigated the trade-off between employing faster, cheaper, yet less sensitive antigen tests at a more frequent rate. When the same testing capacity was used, the antigen tests underperformed the PCR tests with one day delay (not shown). This was due to decreased interval of detection for the antigen tests ($\tau_{1}^{v}=3.2$ and $\tau_{4}^{v}=6.7$ days post infection). If the frequency of antigen test administration was increased, or in places where the PCR return is long, antigen test can present a reliable alternative for surveillance testing.

Lastly, our model assumed that the individuals that received their first vaccine dose and then get infected within the first $\alpha_{v}=11$ days have the same virus profile as a non-vaccinated individual. The results of this study are not sensitive to the value of $\alpha_{v}$ (not shown). In the model, such individuals may then complete vaccination following recovery, which may differ from current public health practices.

## 5. Conclusions

In summary, we have developed a multi-scale model of SARS-CoV-2 transmission in a vaccinated population. We found that, when variants with low transmission rates and for which vaccines are highly efficacious (such as the Alpha variant) are dominant, testing is effective when vaccination levels are low to moderate and its impact is diminished when vaccination levels are high. When variants with increased transmission rates and for which vaccines are less efficacious (such as the Delta variant) are dominant, widespread vaccination is necessary in order to prevent significant outbreaks. When only moderate vaccination level can be achieved, frequent testing can significantly reduce the cumulative size of the outbreak, and the impact of testing is greatest when it is focused on the remaining non-vaccinated population.

## Figures and Tables

**Table 1:** Parameter values and initial conditions used in model Equation (1).

Fixed Parameters	Description	Value	Source
β	Transmission rate	varied	
*b*	Birth rate	$7\times 10^{-4}$/day	[25]
μ	Death rate	$7\times 10^{-4}$/day	[25]
*m*	Disease induced mortality rate	$10^{-3}$/day	
*f*	Fraction of symptomatic infections	0.7	[30]
γ	Relative asymp. infectiousness	0.7	
$\alpha_{v}$	Age of vaccination for reduced virus	11 days	[27]
$\alpha_{f}$	Age of vaccination corresponding to full protection	35 days	[12]
$\tau_{q}$	Recovery age	14 days	[24]
*ℓ*	PCR test return delay	1 day	
$\tau_{1}^{j}$ (j={a,s})	Age of onset of virus detectability in non-vaccinated	0.554 days	
$\tau_{1}^{j}$ (j={va,vs})	Age of onset of virus detectability in vaccinated	0.974 days	
$\tau_{2}^{j}$ (j={a,s})	Age of onset of infectiousness in non-vaccinated	2.5 days	[31]
$\tau_{2}^{j}$ (j={va,vs})	Age of onset of infectiousness in vaccinated	2.8 days	[27]
$\tau_{3}^{j}$ (j={a,s})	Age of end of infectiousness in non-vaccinated	10.5 days	[30,32]
$\tau_{3}^{j}$ (j={va,vs})	Age of end of infectiousness in vaccinated	9.8 days	[27]
$\tau_{4}^{j}$ (j={a,s})	Age of loss of virus detectability in non-vaccinated	10.95 days	
$\tau_{4}^{j}$ (j={va,vs})	Age of loss of virus detectability in vaccinated	10.22 days	
ν	Vaccination rate	varied	
$t_{1}$	Time when additional vaccination is initiated	20 days	
*C*	testing capacity	varied	
**Initial Conditions**	**Description**	**Value**	**Source**
S(0)	Susceptible population	0.95−V(0)	
$V\left(\alpha ,0\right)=V\left(0\right)=V_{0}$	Vaccination level	varied	
$i_{s}\left(\tau ,0\right)$	Infected symptomatic population	0.05fδ(τ)	
$i_{a}\left(\tau ,0\right)$	Infected asymptomatic population	0.05(1−f)δ(τ)	
$i_{vs}\left(\tau ,0\right)$	Infected vaccinated symptomatic population	0	
$i_{va}\left(\tau ,0\right)$	Infected vaccinated asymptomatic population	0	
R(0)	Recovered population	0	
$R_{v1}\left(0\right)$	Vaccinated after natural infection	0	
$R_{v2}\left(0\right)$	Vaccinated, infected, and recovered	0	

## Data Availability

The code generated will be available upon acceptance.

## References

[1] Zhu N., Zhang D., Wang W., Li X., Yang B., Song J., Zhao X., Huang B., Shi W., Lu R. (2020). A novel coronavirus from patients with pneumonia in China, 2019. N. Engl. J. Med..

[2] WHO Cases Dashboard. https://covid19.who.int/.

[3] Legido-Quigley H., Asgari N., Teo Y.Y., Leung G.M., Oshitani H., Fukuda K., Cook A.R., Hsu L.Y., Shibuya K., Heymann D. (2020). Are high-performing health systems resilient against the COVID-19 epidemic?. Lancet.

[4] Rocha R., Atun R., Massuda A., Rache B., Spinola P., Nunes L., Lago M., Castro M.C. (2021). Effect of socioeconomic inequalities and vulnerabilities on health-system preparedness and response to COVID-19 in Brazil: A comprehensive analysis. Lancet Glob. Health.

[5] Haldane V., De Foo C., Abdalla S.M., Jung A.S., Tan M., Wu S., Chua A., Verma M., Shrestha P., Singh S. (2021). Health systems resilience in managing the COVID-19 pandemic: Lessons from 28 countries. Nat. Med..

[6] Ingram C., Downey V., Roe M., Chen Y., Archibald M., Kallas K.A., Kumar J., Naughton P., Uteh C.O., Rojas-Chaves A. (2021). COVID-19 prevention and control measures in workplace settings: A rapid review and meta-analysis. Int. J. Environ. Res. Public Health.

[7] Perra N. (2021). Non-pharmaceutical interventions during the COVID-19 pandemic: A review. Phys. Rep..

[8] Matrajt L., Leung T. (2020). Evaluating the effectiveness of social distancing interventions to delay or flatten the epidemic curve of coronavirus disease. Emerg. Infect. Dis..

[9] Kumar A., Dowling W.E., Román R.G., Chaudhari A., Gurry C., Le T.T., Tollefson S., Clark C.E., Bernasconi V., Kristiansen P.A. (2021). Status report on COVID-19 vaccines development. Curr. Infect. Dis. Rep..

[10] Callaway E. (2020). The race for coronavirus vaccines: A graphical guide. Nature.

[11] Polack F.P., Thomas S.J., Kitchin N., Absalon J., Gurtman A., Lockhart S., Perez J.L., Marc G.P., Moreira E.D., Zerbini C. (2020). Safety and efficacy of the BNT162b2 mRNA Covid-19 vaccine. N. Engl. J. Med..

[12] U.S. COVID-19 Vaccine Product Information. https://www.cdc.gov/vaccines/covid-19/info-by-product/index.html.

[13] Tenforde M.W. (2021). Sustained Effectiveness of Pfizer-BioNTech and Moderna Vaccines Against COVID-19 Associated Hospitalizations Among Adults—United States, March–July 2021. Morb. Mortal. Wkly. Rep..

[14] Bernal J.L., Andrews N., Gower C., Robertson C., Stowe J., Tessier E., Simmons R., Cottrell S., Roberts R., O’Doherty M. (2021). Effectiveness of the Pfizer-BioNTech and Oxford-AstraZeneca vaccines on covid-19 related symptoms, hospital admissions, and mortality in older adults in England: Test negative case-control study. BMJ.

[15] Haas E.J., Angulo F.J., McLaughlin J.M., Anis E., Singer S.R., Khan F., Brooks N., Smaja M., Mircus G., Pan K. (2021). Impact and effectiveness of mRNA BNT162b2 vaccine against SARS-CoV-2 infections and COVID-19 cases, hospitalisations, and deaths following a nationwide vaccination campaign in Israel: An observational study using national surveillance data. Lancet.

[16] Tenforde M.W., Patel M.M., Ginde A.A., Douin D.J., Talbot H.K., Casey J.D., Mohr N.M., Zepeski A., Gaglani M., McNeal T. (2021). Effectiveness of SARS-CoV-2 mRNA vaccines for preventing Covid-19 hospitalizations in the United States. Clin. Infect. Dis..

[17] CDC Covid-19 Variants Proportion in USA. https://covid.cdc.gov/covid-data-tracker/#variant-proportions.

[18] Fowlkes A. (2021). Effectiveness of COVID-19 Vaccines in Preventing SARS-CoV-2 Infection Among Frontline Workers Before and During B. 1.617. 2 (Delta) Variant Predominance—Eight US Locations, December 2020–August 2021. MMWR. Morb. Mortal. Wkly. Rep..

[19] Lopez Bernal J., Andrews N., Gower C., Gallagher E., Simmons R., Thelwall S., Stowe J., Tessier E., Groves N., Dabrera G. (2021). Effectiveness of Covid-19 vaccines against the B.1.617.2 (delta) variant. N. Engl. J. Med..

[20] Liu Y., Rocklöv J. (2021). The reproductive number of the Delta variant of SARS-CoV-2 is far higher compared to the ancestral SARS-CoV-2 virus. J. Travel Med..

[21] Earnest R., Uddin R., Matluk N., Renzette N., Siddle K.J., Loreth C., Adams G., Tomkins-Tinch C., Petrone M.E., Rothman J.E. (2021). Comparative transmissibility of SARS-CoV-2 variants Delta and Alpha in New England, USA. medRxiv.

[22] Birhane M., Bressler S., Chang G., Clark T., Dorough L., Fischer M., Watkins L.F., Goldstein J.M., Kugeler K., Langley G. (2021). COVID-19 Vaccine Breakthrough Infections Reported to CDC—United States, January 1–April 30, 2021. Morb. Mortal. Wkly. Rep..

[23] Pfizer-BioNTech Booster Approval. https://www.fda.gov/news-events/press-announcements/fda-authorizes-booster-dose-pfizer-biontech-covid-19-vaccine-certain-populations.

[24] Forde J., Ciupe S. (2021). Quantification of the tradeoff between test sensitivity and test frequency in a COVID-19 epidemic-a multi-scale modeling approach. Viruses.

[25] Nikin-Beers R., Blackwood J.C., Childs L.M., Ciupe S.M. (2018). Unraveling within-host signatures of dengue infection at the population level. J. Theor. Biol..

[26] Dorratoltaj N., Nikin-Beers R., Ciupe S.M., Eubank S.G., Abbas K.M. (2017). Multi-scale immunoepidemiological modeling of within-host and between-host HIV dynamics: Systematic review of mathematical models. PeerJ.

[27] Levine-Tiefenbrun M., Yelin I., Katz R., Herzel E., Golan Z., Schreiber L., Wolf T., Nadler V., Ben-Tov A., Kuint J. (2021). Initial report of decreased SARS-CoV-2 viral load after inoculation with the BNT162b2 vaccine. Nat. Med..

[28] Pfizer-BioNTech COVID-19 Vaccine EUA. https://www.fda.gov/media/144414/download.

[29] Ke R., Zitzmann C., Ribeiro R.M., Perelson A.S. (2020). Kinetics of SARS-CoV-2 infection in the human upper and lower respiratory tracts and their relationship with infectiousness. medRxiv.

[30] He D., Zhao S., Lin Q., Zhuang Z., Cao P., Wang M.H., Yang L. (2020). The relative transmissibility of asymptomatic COVID-19 infections among close contacts. Int. J. Infect. Dis..

[31] Wölfel R., Corman V.M., Guggemos W., Seilmaier M., Zange S., Müller M.A., Niemeyer D., Jones T.C., Vollmar P., Rothe C. (2020). Virological assessment of hospitalized patients with COVID-2019. Nature.

[32] He X., Lau E.H., Wu P., Deng X., Wang J., Hao X., Lau Y.C., Wong J.Y., Guan Y., Tan X. (2020). Temporal dynamics in viral shedding and transmissibility of COVID-19. Nat. Med..

[33] CDC Guidelines for Vaccinated People. https://www.cdc.gov/coronavirus/2019-ncov/vaccines/fully-vaccinated-guidance.html.

[34] Motta F.C., McGoff K.A., Deckard A., Wolfe C.R., Moody M.A., Cavanaugh K., Denny T.N., Harer J., Haase S.B. (2021). Benefits of Surveillance Testing and Quarantine in a SARS-CoV-2 Vaccinated Population of Students on a University Campus. medRxiv.

[35] Hacisuleyman E., Hale C., Saito Y., Blachere N.E., Bergh M., Conlon E.G., Schaefer-Babajew D.J., DaSilva J., Muecksch F., Gaebler C. (2021). Vaccine breakthrough infections with SARS-CoV-2 variants. N. Engl. J. Med..

[36] Bergwerk M., Gonen T., Lustig Y., Amit S., Lipsitch M., Cohen C., Mandelboim M., Gal Levin E., Rubin C., Indenbaum V. (2021). COVID-19 breakthrough infections in vaccinated health care workers. N. Engl. J. Med..

[37] Duerr R., Dimartino D., Marier C., Zappile P., Wang G., Lighter J., Elbel B., Troxel A.B., Heguy A. (2021). Dominance of Alpha and Iota variants in SARS-CoV-2 vaccine breakthrough infections in New York City. J. Clin. Investig..

[38] Musser J.M., Christensen P.A., Olsen R.J., Long S.W., Subedi S., Davis J.J., Hodjat P., Walley D.R., Kinskey J.C., Gollihar J.D. (2021). Delta variants of SARS-CoV-2 cause significantly increased vaccine breakthrough COVID-19 cases in Houston, Texas. medRxiv.

[39] Luo C.H., Morris C.P., Sachithanandham J., Amadi A., Gaston D., Li M., Swanson N.J., Schwartz M., Klein E.Y., Pekosz A. (2021). Infection with the SARS-CoV-2 Delta Variant is Associated with Higher Infectious Virus Loads Compared to the Alpha Variant in both Unvaccinated and Vaccinated Individuals. medRxiv.

[40] Moline H.L., Whitaker M., Deng L., Rhodes J.C., Milucky J., Pham H., Patel K., Anglin O., Reingold A., Chai S.J. (2021). Effectiveness of COVID-19 vaccines in preventing hospitalization among adults aged ≥ 65 years—COVID-NET, 13 states, February–April 2021. Morb. Mortal. Wkly. Rep..

[41] Rella S.A., Kulikova Y.A., Dermitzakis E.T., Kondrashov F.A. (2021). Rates of SARS-CoV-2 transmission and vaccination impact the fate of vaccine-resistant strains. Sci. Rep..

